# Protective Effects of Cinnamaldehyde against Mesenteric Ischemia-Reperfusion-Induced Lung and Liver Injuries in Rats

**DOI:** 10.1155/2020/4196548

**Published:** 2020-12-09

**Authors:** Marwan Almoiliqy, Jin Wen, Eskandar Qaed, Yuchao Sun, Mengqiao Lian, Haithm Mousa, Mahmoud Al-Azab, Mohamed Y. Zaky, Dapeng Chen, Li Wang, Abdulkarem AL-Sharabi, Zhihao Liu, Pengyuan Sun, Yuan Lin

**Affiliations:** ^1^Department of Pharmacology, Pharmaceutical College, Dalian Medical University, Dalian 116044, China; ^2^Key Lab of Aromatic Plant Resources Exploitation and Utilization in Sichuan Higher Education, Yibin University, Yibin, 644000 Sichuan, China; ^3^Department of Clinical Biochemistry, Dalian Medical University, Dalian 116044, China; ^4^Department of Immunology, Guangzhou Institute of Pediatrics, Guangzhou Women and Children's Medical Center, Guangzhou Medical University, Guangzhou 510623, China; ^5^Institute of Cancer Stem Cell, Dalian Medical University, Dalian 116044, China; ^6^Molecular Physiology Division, Faculty of Science, Beni-Suef University, Beni-Suef, Egypt; ^7^Laboratory Animal Center, Dalian Medical University, Dalian 116044, China

## Abstract

The aim of this study was to characterize and reveal the protective effects of cinnamaldehyde (CA) against mesenteric ischemia-reperfusion- (I/R-) induced lung and liver injuries and the related mechanisms. Sprague-Dawley (SPD) rats were pretreated for three days with 10 or 40 mg/kg/d, ig of CA, and then induced with mesenteric ischemia for 1 h and reperfusion for 2 h. The results indicated that pretreatment with 10 or 40 mg/kg of CA attenuated morphological damage in both lung and liver tissues of mesenteric I/R-injured rats. CA pretreatment significantly restored the levels of aspartate transaminase (AST) and alanine transaminase (ALT) in mesenteric I/R-injured liver tissues, indicating the improvement of hepatic function. CA also significantly attenuated the inflammation via reducing myeloperoxidase (MOP) activity and downregulating the expression of inflammation-related proteins, including interleukin-6 (IL-6), interleukin-1*β* (IL-1*β*), cyclooxygenase-2 (Cox-2), and tumor necrosis factor receptor type-2 (TNFR-2) in both lung and liver tissues of mesenteric I/R-injured rats. Pretreatment with CA significantly downregulated nuclear factor kappa B- (NF-*κ*B-) related protein expressions (NF-*κ*B p65, NF-*κ*B p50, I kappa B alpha (IK-*α*), and inhibitor of nuclear factor kappa-B kinase subunit beta (IKK*β*)) in both lung and liver tissues of mesenteric I/R-injured rats. CA also significantly downregulated the protein expression of p53 family members, including caspase-3, caspase-9, Bax, and p53, and restored Bcl-2 in both lung and liver tissues of mesenteric I/R-injured rats. CA pretreatment significantly reduced TUNEL-apoptotic cells and significantly inhibited p53 and NF-*κ*B p65 nuclear translocation in both lung and liver tissues of mesenteric I/R-injured rats. CA neither induced pulmonary and hepatic histological alterations nor affected the parameters of inflammation and apoptosis in sham rats. We conclude that CA alleviated mesenteric I/R-induced pulmonary and hepatic injuries via attenuating apoptosis and inflammation through inhibition of NF-*κ*B and p53 pathways in rats, suggesting the potential role of CA in remote organ ischemic injury protection.

## 1. Introduction

Mesenteric I/R injury is a serious pathological condition with characteristics of hemorrhagic shock, trauma, strangulated intestinal obstruction, and acute mesenteric ischemia (AMI) [1–3]. Mesenteric I/R injury may induce remote organ injuries, including lung and liver injuries which are associated with high morbidity and mortality [4–6]. Mesenteric I/R-induced pulmonary injury may lead to either acute dysfunction or severe dysfunction (failure), which may further cause myocardial, hepatic, and renal failure followed by death [7–9]. Mesenteric I/R injury is a clinical challenge, and the effective therapeutic strategy is limited with the exception of surgery [10–12], showing the requirement of novel treatment options for ameliorating both direct mesenteric I/R injury and indirect remote organ injuries.

Protective effects induced by natural compounds are found to ameliorate mesenteric I/R-induced local and/or remote organ injuries. For instance, ginsenoside Rb1 ameliorates mesenteric I/R-induced lung injury [13]; curcumin alleviates pulmonary and renal injuries induced by mesenteric I/R, respectively [14, 15]; and ghrelin ameliorates mesenteric I/R-induced lung injury [16]. Irisin protects against mesenteric I/R-induced liver injury [17]. And in our previous study, CA attenuates mesenteric I/R-induced gut injury via a synergistic inhibition of p53/NF-*κ*B signaling pathways [18].

**Figure 1:**
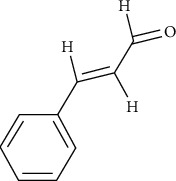
Chemical structure of cinnamaldehyde.

Cinnamaldehyde (CA, Figure 1) is the active constituent of cinnamon extract obtained from the bark of *Cinnamomum* [19, 20]. CA has various beneficial effects, such as antibacterial [21], anti-inflammatory [22], antioxidative [23], and antiapoptotic effects [24]. CA is found to protect against gram-positive/negative infection [25], diabetes [26], gastric ulcer [27], cardiac hypertrophy [28], and myocardial [29]/brain I/R injuries [30, 31]. However, whether CA can efficiently protect against mesenteric I/R-induced injuries in the lung and liver still needs to be revealed. Based on our preexperiments, we proposed that CA pretreatment could ameliorate mesenteric I/R-induced liver and lung injuries via attenuating inflammation and apoptosis through inhibition of both NF-*κ*B and p53 signaling pathways. Rat models of mesenteric I/R-induced lung and liver injuries were used to verify our proposal.

## 2. Materials and Methods

### 2.1. Chemicals and Materials

From Aladdin (Aladdin, Shanghai, China), cinnamaldehyde (purity: ≥98%) was purchased. The assay kits for detecting alanine transaminase (ALT), aspartate transaminase (AST), and myeloperoxidase (MPO) and an assay of terminal deoxynucleotidyl transferase dUTP nick end labeling (TUNEL) were obtained from Nanjing Jiancheng Institute of Biotechnology (Nanjing, Jiangsu, China). The protein extraction kits, bicinchoninic acid protein assay kits, and hematoxylin and eosin staining kits were obtained from Beyotime Institute of Biotechnology (Haimen, Jiangsu, China). 4′,6-Diamidino-2-phenylindole (DAPI) was obtained from Sigma-Aldrich (St. Louis, MO, USA). All other reagents were of analytical grade.

### 2.2. Animals

200-220 g male Sprague-Dawley (SPD) rats were provided by the Animal Center (Dalian Medical University) (certificate of conformity: NO.SCXK (Liao) 2018-0003). According to the National Institutes of Health guideline (publication no. 85-23, revised 1985) and Dalian Medical University (approval number: L20140402) protocols, rats were received care and housed one per cage under daily hygiene and proper environment.

### 2.3. Induction of Mesenteric I/R Model

Mesenteric I/R injury on a rat model was induced as previously described [32, 33]. 12 h before the I/R injury, the animals were fasted of food, and then, on the day of the experiment, the rats were anesthetized with pentobarbital (50 mg/kg of body weight) intraperitoneally (ip). The superior mesenteric artery (SMA) was clamped with a traumatic microvascular clamp for 1 h to achieve ischemia; then, the SMA was unclamped for an additional 2 h to induce reperfusion. And the lung and liver tissue samples were collected and placed on ice, rinsed with phosphate-buffered saline (PBS), and stored at -80°C after the rats were euthanized. Segments of lung and liver tissue were fixed with formalin for TUNEL, immunofluorescence, and histological analysis.

The rats were randomly assigned to 5 groups (5 rats/group): (1) sham group: via the intragastric gavage (ig) route, rats were given a vehicle daily for 3 days before rat sham surgery; (2) sham+CA group: rats were subjected to sham surgery after pretreatment with CA (ig) with the concentration 40 mg/kg daily/3 days; (3) I/R group: via the intragastric gavage (ig) route, rats were given a vehicle daily for 3 days before they were induced with 1 h mesenteric ischemia and then reperfusion for additional 2 h; (4) I/R+CA (L) group: rats were subjected to I/R surgery after they were pretreated with CA (ig) with the concentration 10 mg/kg/day/3 days [18, 34]; and (5) I/R+CA (H) group: rats were subjected to I/R surgery after they were pretreated with CA (ig) with the concentration 40 mg/kg/day/3 days [18, 35, 36]. The intragastric gavage suspension of CA in carboxymethyl cellulose (1% CMC) was prepared daily and freshly and given at 2 mL/kg.

### 2.4. Tissue Staining and Histology

Hematoxylin and eosin (H&E) (H&E staining®, Haimen, Jiangsu, China) staining was performed after the lung and liver tissue samples were fixed in formalin solution, paraffined, and then sliced. The samples were randomly selected and stained with H&E staining according to the manufacturer's instruction (H&E staining®, Haimen, Jiangsu, China). The extent of I/R-induced lung and liver injuries causing histopathological damages was evaluated as prescribed. The lung injury score was evaluated as grades ranging from 0 (minimum) to 4 (maximum), of each of the following 3 terms: accumulation of the inflammatory cells, alveolar wall thickness, and alveolar hemorrhage [37]. The liver histological score was evaluated as grades ranging from 0 (minimum) to 4 (maximum), of each of the following 6 terms: condensation of the nucleus, fragmentation of the nucleus, nuclear fading, cytoplasmic color fading, vacuolization, and erythrocyte stasis [38].

### 2.5. Biochemical Analysis

The levels of tissue ALT, AST, and MPO were measured according to the manufacturer's instructions (Nanjing, Jiangsu, China). At 4°C condition, the rat lung and liver tissues were homogenized in saline and then centrifuged for 10 min at 3000 g/min. Tissue enzyme activities were determined by using the assay kits (Nanjing, Jiangsu, China).

### 2.6. Immunofluorescence Staining

Paraffin-embedded rat lung and liver tissue slides were dewaxed, and 0.1% TritonX-100 solution was added for 10 min, and then, the slides were washed with PBS three times. Tissue samples were incubated with primary antibodies against NF-*κ*B p65 and p53 (Proteintech, Wuhan, Hubei, China; 1 : 100) overnight at 4°C, then incubated with secondary antibody (Proteintech, Wuhan, Hubei, China; 1 : 100), and then, the slides were washed with PBS and stained with DAPI (1 *μ*g/mL). Samples were visualized using fluorescence microscopy (BX63, IX81, Olympus, Japan).

### 2.7. Immunostaining of TUNEL

Rat lung and liver tissue slides were dewaxed with gradients of alcohol, then permeabilized with 0.1% TritonX-100 solution for 10 min; after that, the slides were washed with PBS. And slides were stained using a TUNEL staining kit (One-Step TUNEL kit®, Nanjing, Jiangsu, China) according to the manufacturer's instruction. The slides were visualized using a fluorescence microscope (BX63, IX81, Olympus, Japan).

### 2.8. Western Blotting

The protein samples of randomly 3 individual rat lung and liver tissues were loaded and resolved using 8, 10, or 12% sodium dodecyl sulfate-polyacrylamide gel electrophoresis (SDS-PAGE), and then, the proteins were transferred to nitrocellulose membranes for 1 h, and at the solution of 5% skimmed milk, the membranes were blocked for an additional 1 h at 37°C. After that, they were incubated with the primary antibody overnight at 4°C: Cox-2, IL-1*β*, IL-6, TNFR-2, caspase-3, caspase-9, Bcl-2, Bax, p53, NF-*κ*B p65, NF-*κ*B p50, IK-*α*, and IKK*β* (Proteintech, Wuhan, Hubei, China). Then, the membranes were washed in Tween-20 and Tris-buffered saline (T-TBS)/3 times and incubated at 37°C in secondary antibody (Proteintech, Wuhan, Hubei, China) for 1 h. Enhanced chemiluminescent (ECL) solution (Proteintech, Wuhan, Hubei, China) was used to visualize the membranes. And proteins were quantified by using Image Lab software (Bio-Rad, CA, USA) for 3 independent experiments. *β*-Actin was the corresponding expression of normalization [39, 40].

### 2.9. Statistical Analysis

One-way analysis of variance (ANOVA) followed by the Student-Newman-Keuls test was used to the normal distribution data. All values are presented as the mean ± standard deviation (SD), of at least 3 independent experiments. Prism 5.0 (GraphPad, La Jolla, CA) software was used for data analysis. *P* values of less than 0.05 indicated the statistical significance of the differences.

## 3. Results

### 3.1. Protective Effects of CA against Mesenteric I/R-Induced Lung and Liver Morphological Damages

**Figure 2:**
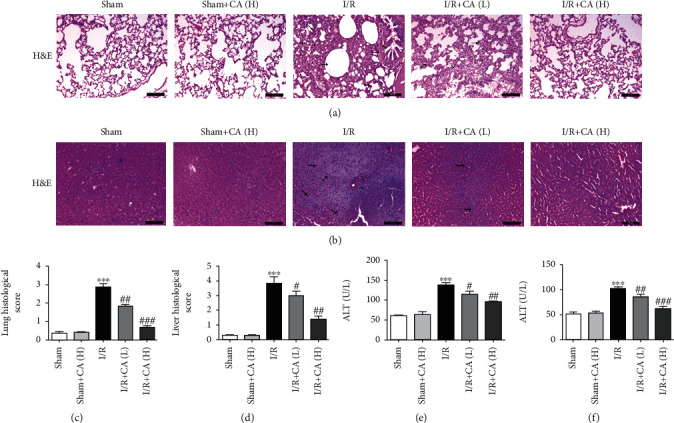
Cinnamaldehyde alleviated morphological damages of both lung and liver tissues of mesenteric I/R-injured rats. (a) Tissue histology images of lung (scale bar = 200 *μ*m; magnification ×100 of H&E). (b) Tissue histology images of liver (scale bar = 200 *μ*m; magnification ×100 of H&E). (c) Histological evaluation of the lung tissues after mesenteric I/R. (d) Histological evaluation of the liver tissues after mesenteric I/R. (e) Tissue level of hepatic ALT. (f) Tissue level of hepatic AST. All results are analyzed as the mean ± SD (*n* = 5). ^∗∗∗^*P* < 0.001*vs.* sham group; ^###^*P* < 0.001, ^##^*P* < 0.01, and ^#^*P* < 0.05*vs.* I/R group.

The following are the pulmonary and hepatic morphological damages induced by mesenteric I/R injury in I/R rats compared with sham rats using H&E staining: characterized with significant inflammatory cell infiltration, perivascular and interstitial edema, deposition in the alveolar spaces, and hemorrhage in lung tissues (Figure 2(a)) and nuclear condensation, cell shrinkage, and margination and apoptotic debris in liver tissues (Figure 2(b)). And these morphological alterations were significantly ameliorated by CA pretreatment at concentrations 10 and 40 mg/kg (Figures 2(a) and 2(b)). The pulmonary and hepatic histopathological scores were significantly increased in injured rats by mesenteric I/R compared with the corresponding sham groups, and pretreatment with CA significantly reduced these elevated scores (Figures 2(c) and 2(d)). CA did not cause any significant pulmonary and hepatic morphological alterations in the sham groups. And mesenteric I/R showed a significant elevation of enzymatic levels of ALT and AST in hepatic tissues of mesenteric I/R-injured rats, showing mesenteric I/R-induced liver damage and hepatic dysfunction compared with sham rats (Figures 2(e) and 2(f)). CA pretreatment induced a significant reduction of the enzymatic activities in the hepatic tissue of the mesenteric I/R-injured rats. CA did not significantly alter ALT and AST levels in sham rats.

### 3.2. Protective Effects of CA against Mesenteric I/R-Induced Inflammation in Lung and Liver Tissues

**Figure 3:**
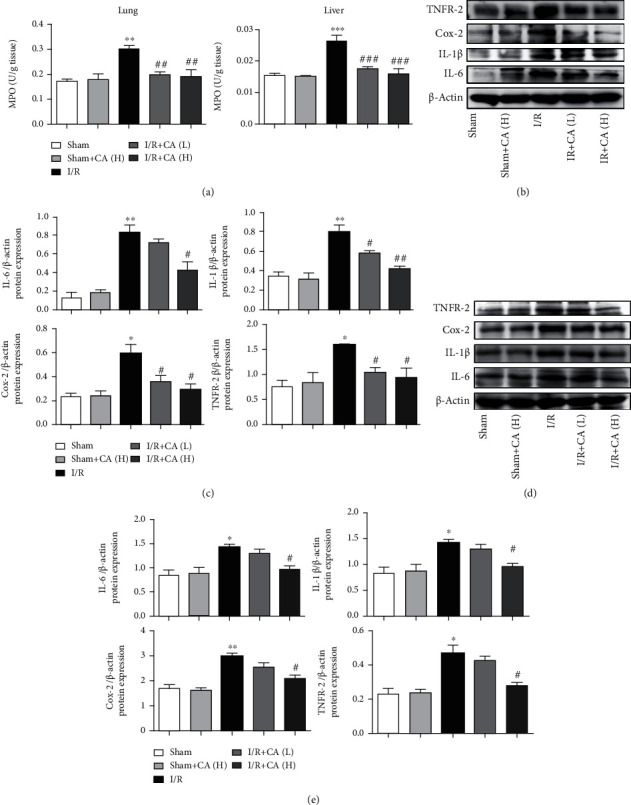
Cinnamaldehyde ameliorated against mesenteric I/R-mediated lung and liver inflammation. (a) Tissue levels of pulmonary and hepatic MPO. (b) The levels of IL-6, IL-1*β*, Cox-2, and TNFR-2 protein expression in lung tissues. (c) Inflammatory protein expression quantifications in lung tissues. (d) The levels of IL-6, IL-1*β*, Cox-2, and TNFR-2 protein expression in liver tissues. (e) Inflammatory protein expression quantifications in liver tissues. All results are analyzed as the mean ± SD (*n* = 3). ^∗∗∗^*P* < 0.001, ^∗∗^*P* < 0.01, and ^∗^*P* < 0.05*vs.* sham group; ^###^*P* < 0.001, ^##^*P* < 0.01, and ^#^*P* < 0.05*vs.* I/R group.

The contents of myeloperoxidase (MPO) in lung and liver tissues were elevated in mesenteric I/R-injured rats significantly compared with the sham rats, and pretreatment with CA at concentrations 10 and 40 mg/kg showed a significant reversion of the increased MPO (Figure 3(a)). The results of western blot indicated that inflammatory protein expressions of IL-6, IL-1*β*, Cox-2, and TNFR-2 were significantly upregulated in both pulmonary and hepatic tissues of the mesenteric I/R-injured rats (Figures 3(b)–3(e)). And CA pretreatment showed a statistically obvious reduction of IL-6, IL-1*β*, Cox-2, and TNFR-2 protein expressions (Figures 3(b)–3(e)), suggesting that CA alleviates mesenteric I/R-induced injuries through attenuating the inflammation. CA did not significantly change these protein expressions in sham rats.

### 3.3. Protective Effects of CA against Mesenteric I/R-Induced Lung and Liver Apoptosis

**Figure 4:**
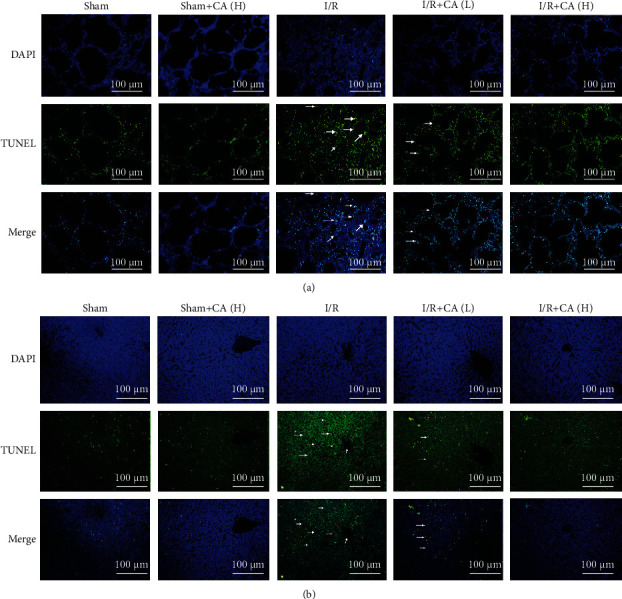
Cinnamaldehyde attenuated apoptosis in both lung and liver tissues of mesenteric I/R-injured rats. (a) TUNEL-apoptotic cell assay in the injured lung after mesenteric I/R (scale bar = 100 *μ*m; magnification ×200). (b) TUNEL-apoptotic cell assay in the injured liver after mesenteric I/R (scale bar = 100 *μ*m; magnification ×200).

Our results indicated that TUNEL-positive apoptotic cells were significantly more observed in both injured pulmonary and hepatic tissues of the mesenteric I/R group compared with the sham groups. And pretreatment with CA significantly decreased TUNEL-positive cells in lung and liver tissues compared with I/R-injured rats (Figures 4(a) and 4(b)), suggesting that pretreatment with CA significantly alleviated mesenteric I/R-induced lung and liver apoptosis. CA did not significantly show TUNEL-positive cells in lung and liver tissues in sham rats.

### 3.4. The Role of CA-Induced Protection against p53 and NF-*κ*B

#### 3.4.1. Cinnamaldehyde Inhibits p53

**Figure 5:**
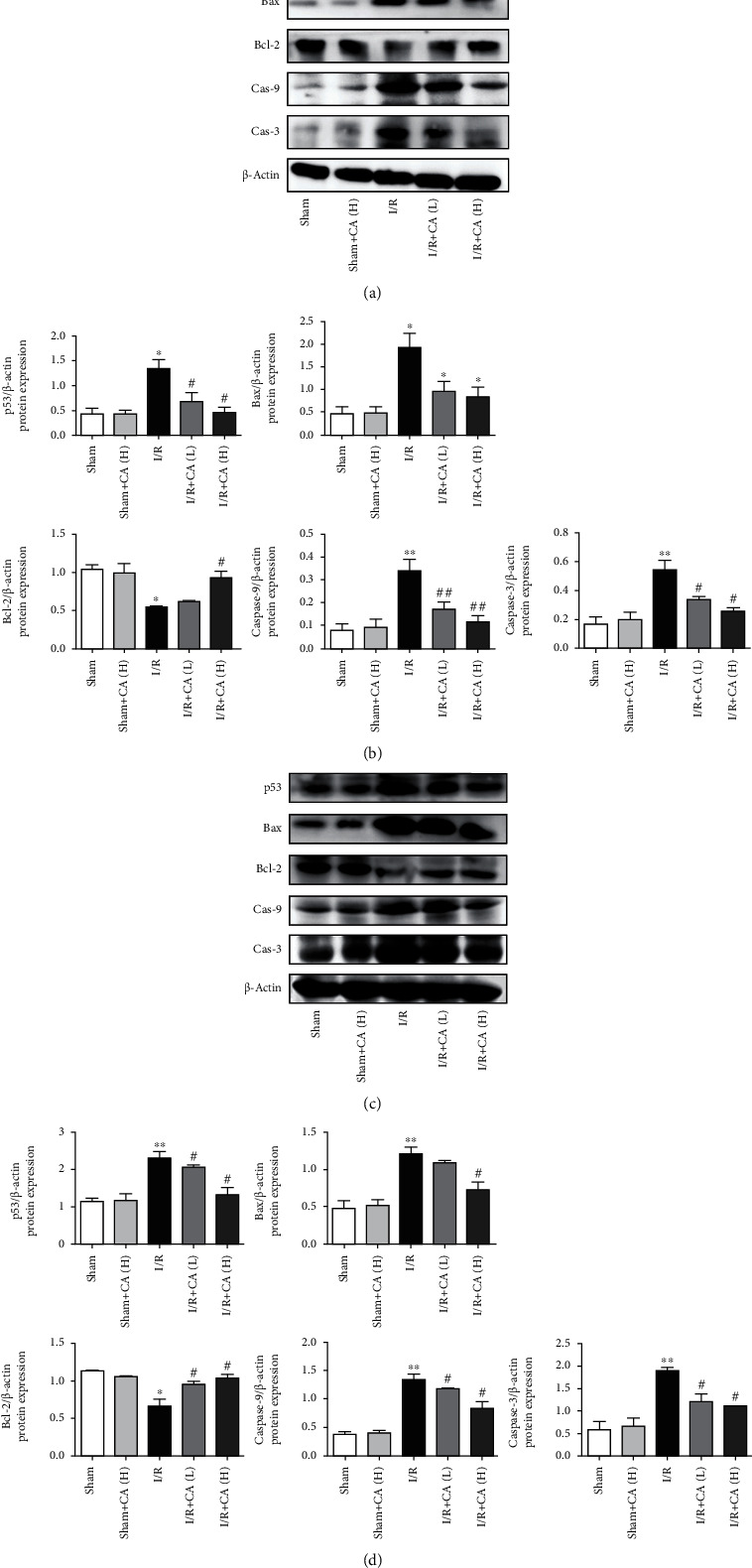
Cinnamaldehyde protected against mesenteric I/R-induced lung and liver injuries through inhibition of p53 in rats. (a) The protein expression levels of caspase-3, caspase-9, Bcl-2, Bax, and p53 in lung tissues of I/R-injured and sham rats. (b) p53-apoptotic protein expression quantifications in lung tissues. (c) The protein expression levels of caspase-3, caspase-9, Bcl-2, Bax, and p53 in liver tissues of I/R-injured and sham rats. (d) p53-apoptotic protein expression quantifications in liver tissues. All results are expressed as the mean ± SD (*n* = 3). ^∗∗^*P* < 0.01 and ^∗^*P* < 0.05*vs.* sham group; ^##^*P* < 0.01 and ^#^*P* < 0.05*vs.* I/R group.

The transcriptional factor p53 is a proapoptotic factor which exerts a crucial role in mediating apoptosis in lung and liver injuries [41, 42]. The results of western blot showed that the expression of apoptosis proteins, such as caspase-3, caspase-9, Bax, and p53, was significantly increased and the antiapoptotic protein expression of Bcl-2 was significantly decreased in both lung and liver tissues of mesenteric I/R rats compared with the sham rats. The pretreatment with CA restored the increased expression of caspase-3, caspase-9, Bax, and p53 and also restored the reduced Bcl-2 significantly in both pulmonary and hepatic tissues of mesenteric I/R-injured rats, suggesting that CA ameliorated mesenteric I/R-induced lung and liver apoptosis (Figures 5(a)–5(d)). CA did not significantly show any changes in the apoptotic protein expressions in sham rats.

#### 3.4.2. Cinnamaldehyde Inhibits NF-*κ*B

**Figure 6:**
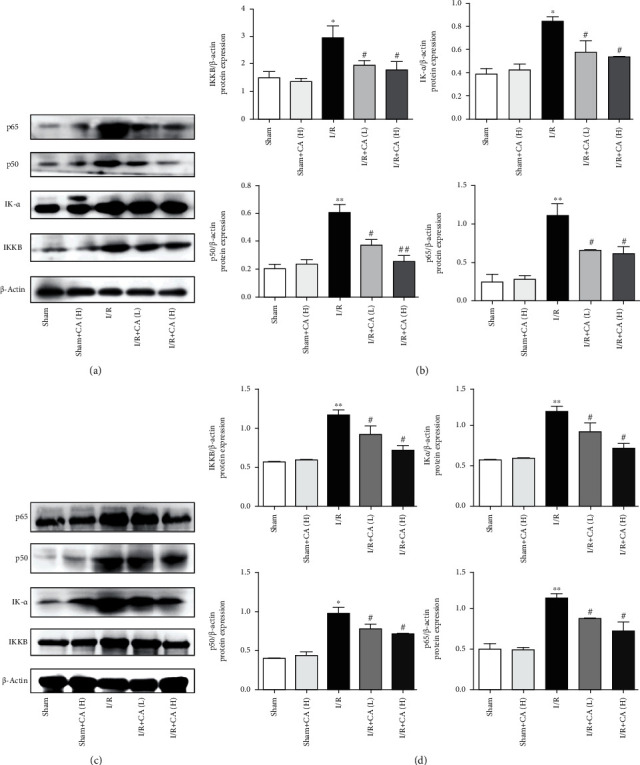
Cinnamaldehyde protected against mesenteric I/R-induced lung and liver injuries by suppression of the NF-*κ*B pathway in rats. (a) The protein expression levels of NF-*κ*B p65, NF-*κ*B p50, IK-*α*, and IKK*β* in lung tissues. (b) NF-*κ*B-related protein quantifications in lung tissues. (c) The protein expression levels of NF-*κ*B p65, NF-*κ*B p50, IK-*α*, and IKK*β* in liver tissues. (d) NF-*κ*B-related protein quantifications in liver tissue. All results are expressed as the mean ± SD (*n* = 3). ^∗∗^*P* < 0.01 and ^∗^*P* < 0.05*vs.* sham group; ^##^*P* < 0.01 and ^#^*P* < 0.05*vs.* I/R group.

The transcriptional factor NF-*κ*B is related to inflammation, immune responses, oxidative stress, and cell death in injured tissues [43–45]. The main NF-*κ*B complex family member is NF-*κ*B p65 [46]. Western blot results showed that NF-*κ*B p65, NF-*κ*B p50, IK-*α*, and IKK*β* protein expressions of NF-*κ*B-related signaling pathway were upregulated in both injured pulmonary and hepatic tissues of mesenteric I/R rats significantly in comparison with the normal tissue controls, and CA significantly downregulated these protein expressions in both injured pulmonary and hepatic tissues of mesenteric I/R rats (Figures 6(a)–6(d)), indicating that CA pretreatment protects against mesenteric I/R-induced lung and liver inflammation and apoptosis. CA did not show any significant protein expression alterations in sham groups.

#### 3.4.3. Cinnamaldehyde Protects against Mesenteric I/R-Triggered p53 and NF-*κ*B p65 Nuclear Translocation in Lung and Liver Tissues

**Figure 7:**
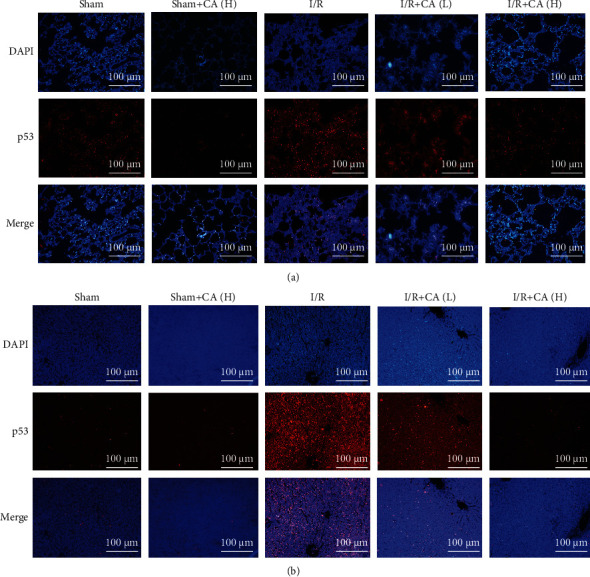
Protective effects of cinnamaldehyde against p53 nuclear translocation in both lung and liver tissues of mesenteric I/R-injured rats. (a) p53 immunofluorescence in sham/I/R-injured lung tissues (scale bar = 100 *μ*m; magnification ×200). (b) p53 immunofluorescence in sham/I/R-injured liver tissues (scale bar = 100 *μ*m; magnification ×200).

The transcriptional factors NF-*κ*B p65 and p53 are both activated under stress conditions, inducing NF-*κ*B p65 and p53 subunit import into the nucleus and triggering the inflammatory mediators and proapoptotic targets [47, 48]. The immunofluorescence assay showed that the p53 nuclear import was more increased in both injured lung and liver tissues of I/R rats compared with the sham animals. And pretreatment with CA inhibited the nuclear translocation of p53 significantly in both injured lung and liver tissues of mesenteric I/R rats (Figures 7(a) and 7(b)), suggesting the role of p53 in mediating CA-induced protection against mesenteric I/R-induced pulmonary and hepatic injuries.

**Figure 8:**
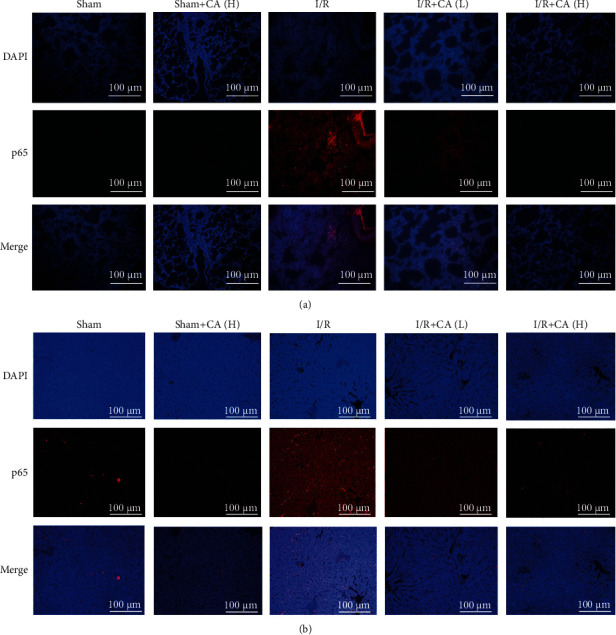
Protective effects of cinnamaldehyde against NF-*κ*B p65 nuclear translocation in both lung and liver tissues of mesenteric I/R-injured rats. (a) NF-*κ*B p65 immunofluorescence in lung tissues of sham/I/R rats (scale bar = 100 *μ*m; magnification ×200). (b) NF-*κ*B p65 immunofluorescence in liver tissues of sham/I/R rats (scale bar = 100 *μ*m; magnification ×200).

The immunofluorescence results also showed that NF-*κ*B p65 nuclear import was also significantly increased in both injured lung and liver tissues in mesenteric I/R rats in comparison with the control groups. And pretreatment with CA inhibited the nuclear translocation of NF-*κ*B p65 significantly in both lung and liver of I/R-injured rats (Figures 8(a) and 8(b)), suggesting the way of NF-*κ*B p65 in mediating CA-induced protection against mesenteric I/R-induced pulmonary and hepatic injuries.

## 4. Discussion

Mesenteric I/R induces either local [49–51] or remote organ injuries, including heart [52], lung [53], liver [54], kidney [55], and brain [56] injuries. Remote organ injuries are a serious consequence of mesenteric I/R injuries due to the damage of intestinal mucosa and translocation of bacteria and endotoxins into the distant body organs [57, 58].

Our previous study showed that mesenteric I/R induced excessive intestinal (local) morphological changes, inflammation, oxidative stress, apoptosis, and upregulated p53 and NF-*κ*B pathway-related proteins in mesenteric I/R-injured rats and hypoxia/reoxygenation- (H/R-) injured intestinal epithelial cells-6 (IEC-6), and CA pretreatment significantly restored all the above-mentioned changes in mesenteric I/R-treated rats and H/R-treated IEC-6 cells [18].

In this study, our results supported our proposal. CA pretreatment alleviated morphological damages in both injured lung and liver tissues of mesenteric I/R rats and significantly restored the injury-related enzymatic alterations of ALT and AST in liver tissues. CA pretreatment significantly attenuated inflammation in mesenteric I/R-induced lung and liver injuries via downregulating the expression of inflammation-related proteins, including IL-6, IL-1*β*, Cox-2, and TNFR-2, and by reversing MPO activity in both injured lung and liver tissues of mesenteric I/R rats.

The transcriptional factors p53 and NF-*κ*B were involved in various tissue injuries [59–62]. NF-*κ*B 65 is the key subunit of the NF-*κ*B pathway (NF-*κ*B p65, NF-*κ*B p50, IK-*α*, and IKK*β*) which creates a crucial role in inducing apoptosis, immune response, and inflammation [63, 64]. And p53 is also the key subunit of the p53 signaling pathway [65]. Our results showed that NF-*κ*B-related proteins, including NF-*κ*B p65, NF-*κ*B p50, IK-*α*, and IKK*β*, were significantly upregulated, and p53-related proteins, including caspase-3, caspase-9, Bax, and p53, were also significantly upregulated, and Bcl-2 was significantly downregulated, in both lung and liver tissues of mesenteric I/R-injured rats. Pretreatment with CA restored these aberrant parameters significantly in both lung and liver tissues of mesenteric I/R-injured rats. Pretreatment with CA showed a significant reduction of TUNEL-apoptotic cells in both injured lung and liver tissues of mesenteric I/R rats, suggesting CA-mediated attenuation of inflammation and apoptosis against mesenteric I/R-induced lung and liver injuries. The immunofluorescence assay showed that CA mediated a significant inhibition of both NF-*κ*B p65 and p53 nuclear translocation in both injured lung and liver tissues of mesenteric I/R rats, indicating that attenuation of inflammation and apoptosis and inhibition of nuclear translocation were related to CA-mediated amelioration against mesenteric I/R-induced lung and liver injuries.

Infection and inflammation are among the major clinical challenges in the treatment of mesenteric I/R injury and are involved in the recommendations for the management and treatment of mesenteric ischemia (acute or chronic) in the recent clinical guidelines [66–70], indicating that both anti-infection and anti-inflammatory interventions are required for treatment of mesenteric I/R-induced local and/or remote organ injuries [71–73]. Although the antibacterial properties of antibiotics are necessary for the treatment of mesenteric I/R, they often cause renal and hepatic injuries [74–76]. Current evidence indicates that CA not only possess ameliorative effects and anti-inflammatory effects for ameliorating tissue injuries but also possess antibacterial activities [25, 77, 78], suggesting that CA could be a potential therapeutic intervention for treating mesenteric I/R-induced local and/or remote organ injuries.

In conclusion, in this study, our results revealed that pretreatment with CA significantly ameliorated and protected against mesenteric I/R-induced lung and liver injuries via reducing aberrant inflammation and apoptosis. CA did not show any significant changes on the corresponding controls. And CA-mediated protection and amelioration against mesenteric I/R-induced remote organ injuries via suppression of p53 and NF-*κ*B exert an important role in the protection. Based on the ameliorative effects together and its bacterial inhibitory effects, this study reveals that CA can be considered a potential choice for alleviating mesenteric I/R-induced remote organ injuries, and the actual relationship of p53 and NF-*κ*B under I/R and remote organ injuries may require future study.

## Data Availability

The data used to support the findings of this study are available from Marwan Almoiliqy, Pengyuan Sun, and Yuan Lin upon request.

## Abbreviations

I/R: Ischemia-reperfusion
H/R: Hypoxia/reoxygenation
CA: Cinnamaldehyde
SMA: Superior mesenteric artery
AMI: Acute mesenteric ischemia
ig: Intragastric gavage
ip: Intraperitoneally
AST: Aspartate transaminase
ALT: Alanine transaminase
H&E: Hematoxylin and eosin
MPO: Myeloperoxidase
TNFR-2: Tumor necrosis factor receptor type-2
Cox-2: Cyclooxygenase-2
IL-6: Interleukin-6
IL-1*β*: Interleukin-1*β*
NF-*κ*B: Nuclear factor kappa B
IK-*α*: I kappa B alpha
IKK*β*: Inhibitor of nuclear factor kappa-B kinase subunit beta
p53: Tumor protein p53
Bax: Bcl-2-associated X protein
Bcl-2: B-cell lymphoma 2
PBS: Phosphate-buffered saline
SPD: Sprague-Dawley
IEC-6: Intestinal epithelial cells-6
SDS-PAGE: Sodium dodecyl sulfate-polyacrylamide gel electrophoresis
T-TBS: Tween-20 and Tris-buffered saline
ECL: Enhanced chemiluminescent
ANOVA: Analysis of variance
SD: Standard deviation
DAPI: 4′,6-Diamidino-2-phenylindole
BCA: Bicinchoninic acid
TUNEL: Terminal deoxynucleotidyl transferase dUTP nick end labeling.

## Additional Points

*Highlights*. (1) Intestinal ischemia/reperfusion-induced lung injury. (2) Intestinal ischemia/reperfusion-induced liver injury. (3) Intestinal ischemia/reperfusion-induced lung and liver inflammation. (4) Intestinal ischemia/reperfusion-induced lung and liver apoptosis. (5) Cinnamaldehyde protects against intestinal ischemia/reperfusion-induced lung and liver inflammation and apoptosis. (6) Cinnamaldehyde protects against intestinal ischemia/reperfusion-induced lung and liver injuries via inhibition of NF-*κ*B and p53 signaling pathways.

## References

[1] Zhao L., Luo L., Chen J., Jia W., Xiao Y., Xiao Y. (2014). Utilization of extracorporeal membrane oxygenation alleviates intestinal ischemia-reperfusion injury in prolonged hemorrhagic shock animal model. *Cell Biochemistry and Biophysics*.

[2] Akbari G. (2020). Emerging roles of microRNAs in intestinal ischemia/reperfusion-induced injury: a review. *Journal of Physiology and Biochemistry*.

[3] Li Y., Wen S., Yao X. (2017). MicroRNA-378 protects against intestinal ischemia/reperfusion injury via a mechanism involving the inhibition of intestinal mucosal cell apoptosis. *Cell Death & Disease*.

[4] Radhakrishnan R. S., Radhakrishnan G. L., Radhakrishnan H. R. (2008). Pretreatment with bone morphogenetic protein-7 (BMP-7) mimics ischemia preconditioning following intestinal ischemia/reperfusion injury in the intestine and liver. *Shock*.

[5] Idrovo J.-P., Yang W.-L., Jacob A. (2014). AICAR attenuates organ injury and inflammatory response after intestinal ischemia and reperfusion. *Molecular Medicine*.

[6] Chen L. W., Egan L., Li Z. W., Kagnoff M. F., Karin M., Karin M. (2003). The two faces of IKK and NF-*κ*B inhibition: prevention of systemic inflammation but increased local injury following intestinal ischemia-reperfusion. *Nature Medicine*.

[7] Murray J. F., Matthay M. A., Luce J. M., Flick M. R. (1988). An expanded definition of the adult respiratory distress syndrome. *American Review of Respiratory Disease*.

[8] Turnage R. H., Guice K. S., Oldham K. T. (1994). Pulmonary microvascular injury following intestinal ischemia/reperfusion: Requirement for neutrophil rolling. *New Horizons*.

[9] Nadatani Y., Watanabe T., Shimada S., Otani K., Tanigawa T., Fujiwara Y. (2017). Neutrophil macroaggregates promote widespread pulmonary thrombosis after gut ischemia. *Science Translational Medicine*.

[10] Nadatani Y., Watanabe T., Shimada S., Tanigawa T., Fujiwara Y., Fujiwara Y. (2018). Microbiome and intestinal ischemia/reperfusion injury. *Journal of Clinical Biochemistry and Nutrition*.

[11] Bertoni S., Ballabeni V., Barocelli E., Tognolini M. (2018). Mesenteric ischemia-reperfusion: an overview of preclinical drug strategies. *Drug Discovery Today*.

[12] Denning N. L., Aziz M., Ochani M., Wang P., Wang P. (2020). Inhibition of a triggering receptor expressed on myeloid cells-1 (TREM-1) with an extracellular cold-inducible RNA-binding protein (eCIRP)-derived peptide protects mice from intestinal ischemia-reperfusion injury. *Surgery*.

[13] Wang J., Qiao L., Li S., Yang G. (2013). Protective effect of ginsenoside Rb1 against lung injury induced by intestinal ischemia-reperfusion in rats. *Molecules (Basel, Switzerland)*.

[14] Fan Z., Yao J., Li Y., Hu X., Shao H., Tian X. (2015). Anti-inflammatory and antioxidant effects of curcumin on acute lung injury in a rodent model of intestinal ischemia reperfusion by inhibiting the pathway of NF-kb. *International Journal of Clinical and Experimental Pathology*.

[15] Onder A., Kapan M., Gumus M. (2012). The protective effects of curcumin on intestine and remote organs against mesenteric ischemia/reperfusion injury. *The Turkish journal of gastroenterology : the official journal of Turkish Society of Gastroenterology*.

[16] Şen L. S., Karakoyun B., Yeğen C. (2015). Treatment with either obestatin or ghrelin attenuates mesenteric ischemia-reperfusion-induced oxidative injury of the ileum and the remote organ lung. *Peptides*.

[17] Fan X., Du J., Wang M.-H. (2019). Irisin contributes to the hepatoprotection of dexmedetomidine during intestinal ischemia/reperfusion. *Oxidative Medicine and Cellular Longevity*.

[18] Almoiliqy M., Wen J., Xu B. (2020). Cinnamaldehyde protects against rat intestinal ischemia/reperfusion injuries by synergistic inhibition of NF-*κ*B and p53. *Acta Pharmacologica Sinica*.

[19] Chanda N., Shukla R., Zambre A. (2011). An effective strategy for the synthesis of biocompatible gold nanoparticles using cinnamon phytochemicals for phantom CT imaging and photoacoustic detection of cancerous cells. *Pharmaceutical Research*.

[20] Lee H.-G., Jo Y., Ameer K., Kwon J. H. (2018). Optimization of green extraction methods for cinnamic acid and cinnamaldehyde from cinnamon (Cinnamomum cassia) by response surface methodology. *Food Science and Biotechnology*.

[21] Sawicki R., Golus J., Przekora A., Ludwiczuk A., Sieniawska E., Ginalska G. (2018). Antimycobacterial activity of cinnamaldehyde in a Mycobacterium tuberculosis(H37Ra) model. *Molecules (Basel, Switzerland)*.

[22] Zhao H., Zhang M., Zhou F. (2016). Cinnamaldehyde ameliorates LPS-induced cardiac dysfunction via TLR4-NOX4 pathway: the regulation of autophagy and ROS production. *Journal of Molecular and Cellular Cardiology*.

[23] Tanaka Y., Uchi H., Furue M. (2019). Antioxidant cinnamaldehyde attenuates UVB-induced photoaging. *Journal of Dermatological Science*.

[24] El-ezz D. A., Maher A., Sallam N., El-brairy A., Kenawy S. (2018). Trans-cinnamaldehyde modulates hippocampal Nrf2 factor and inhibits amyloid beta aggregation in LPS-induced neuroinflammation mouse model. *Neurochemical Research*.

[25] Doyle A. A., Stephens J. C. (2019). A review of cinnamaldehyde and its derivatives as antibacterial agents. *Fitoterapia*.

[26] Zhu R., Liu H., Liu C. (2017). Cinnamaldehyde in diabetes: a review of pharmacology, pharmacokinetics and safety. *Pharmacological Research*.

[27] Tankam J. M., Sawada Y., Ito M. (2013). Regular ingestion of cinnamomi cortex pulveratus offers gastroprotective activity in mice. *Journal of Natural Medicines*.

[28] Yang L., Wu Q.-Q., Liu Y., Hu Z. F., Bian Z. Y., Tang Q. Z. (2015). Cinnamaldehyde attenuates pressure overload-induced cardiac hypertrophy. *International Journal of Clinical and Experimental Pathology*.

[29] Sedighi M., Nazari A., Faghihi M. (2018). Protective effects of cinnamon bark extract against ischemia-reperfusion injury and arrhythmias in rat. *Phytotherapy research : PTR*.

[30] Qi X., Zhou R., Liu Y. (2016). Trans-cinnamaldehyde protected PC12 cells against oxygen and glucose deprivation/reperfusion (OGD/R)-induced injury via anti-apoptosis and anti-oxidative stress. *Molecular and Cellular Biochemistry*.

[31] Chen Y.-F., Wang Y.-W., Huang W.-S. (2016). Trans-cinnamaldehyde, an essential oil in cinnamon powder, ameliorates cerebral ischemia-induced brain injury via inhibition of neuroinflammation through attenuation of iNOS, COX-2 expression and NF*κ*-B signaling pathway. *Neuromolecular Medicine*.

[32] Sun Y., Lian M., Lin Y. (2018). Role of p-MKK7 in myricetin-induced protection against intestinal ischemia/reperfusion injury. *Pharmacological Research*.

[33] Wen J., Xu B., Sun Y. (2019). Paeoniflorin protects against intestinal ischemia/reperfusion by activating LKB1/AMPK and promoting autophagy. *Pharmacological Research*.

[34] Yang L., Wu Q. Q., Liu Y., Hu Z. -F., Bian Z. -Y., Tang Q. -Z. (2015). Cinnamaldehyde attenuates pressure overload-induced cardiac hypertrophy. *International Journal of Clinical and Experimental Pathology*.

[35] Song F., Li H., Sun J., Wang S. (2013). Protective effects of cinnamic acid and cinnamic aldehyde on isoproterenol-induced acute myocardial ischemia in rats. *Journal of Ethnopharmacology*.

[36] Zhao J., Zhang X., Dong L. (2015). Cinnamaldehyde inhibits inflammation and brain damage in a mouse model of permanent cerebral ischaemia. *British Journal of Pharmacology*.

[37] Maltesen R., Buggeskov K., Andersen C. (2018). Lung protection strategies during cardiopulmonary bypass affect the composition of bronchoalveolar fluid and lung tissue in cardiac surgery patients. *Metabolites*.

[38] Yang F., Cui R., Li Z. (2019). Methane alleviates acetaminophen-induced liver injury by inhibiting inflammation, oxidative stress, endoplasmic reticulum stress, and apoptosis through the Nrf2/HO-1/NQO1 signaling pathway. *Oxidative Medicine and Cellular Longevity*.

[39] Alam R., Schultz C. R., Golembieski W. A., Poisson L. M., Rempel S. A. (2013). PTEN suppresses SPARC-induced pMAPKAPK2 and inhibits SPARC-induced Ser78 HSP27 phosphorylation in glioma. *Neuro-Oncology*.

[40] Crane J. D., Ogborn D. I., Cupido C. (2012). Massage therapy attenuates inflammatory signaling after exercise-induced muscle damage. *Science Translational Medicine*.

[41] Li M., Lu Y., Hu Y. (2014). Salvianolic acid B protects against acute ethanol-induced liver injury through SIRT1-mediated deacetylation of p53 in rats. *Toxicology Letters*.

[42] Tousson E., Hafez E., Zaki S., Gad A. (2014). p53, Bcl-2 and CD68 expression in response to amethopterin-induced lung injury and ameliorating role of l-carnitine. *Biomedicine and Pharmacotherapy*.

[43] Yu Y., Zhang L., Liu Q., Lin T., Sun H., Guo H. (2015). Endoplasmic reticulum stress preconditioning antagonizes low-density lipoprotein-induced inflammation in human mesangial cells through upregulation of XBP1 and suppression of the IRE1*α*/IKK/NF-*κ*B pathway. *Molecular Medicine Reports*.

[44] Cau S. B. A., Guimaraes D. A., Rizzi E., Ceron C. S., Gerlach R. F., Tanus-Santos J. E. (2015). The nuclear factor kappaB inhibitor pyrrolidine dithiocarbamate prevents cardiac remodelling and matrix metalloproteinase-2 up-regulation in renovascular hypertension. *Basic & Clinical Pharmacology & Toxicology*.

[45] Yang J., Wang C., Nie X. (2015). Perfluorooctane sulfonate mediates microglial activation and secretion of TNF-*α* through Ca^2+^-dependent PKC-NF-кB signaling. *International Immunopharmacology*.

[46] Epinat J.-C., Whiteside S. T., Rice N. R., Israël A. (1997). Reconstitution of the NF-*κ*B system inSaccharomyces cerevisiae for isolation of effectors by phenotype modulation. *Yeast (Chichester, England)*.

[47] Tamura R., Morimoto K., Hirano S. (2014). Santonin-related compound 2 inhibits the nuclear translocation of NF-*κ*B subunit p65 by targeting cysteine 38 in TNF-*α*-induced NF-*κ*B signaling pathway. *Bioscience, Biotechnology, and Biochemistry*.

[48] Guay D., Gaudreault I., Massip L., Lebel M. (2006). Formation of a nuclear complex containing the p53 tumor suppressor, YB-1, and the Werner syndrome gene product in cells treated with UV light. *The International Journal of Biochemistry & Cell Biology*.

[49] Gurley B. J., Barone G. W., Yamashita K., Polston S., Estes M., Harden A. (1997). Extrahepatic ischemia-reperfusion injury reduces hepatic oxidative drug metabolism as determined by serial antipyrine clearance. *Pharmaceutical Research*.

[50] Li Y., Xu B., Xu M. (2017). 6-Gingerol protects intestinal barrier from ischemia/reperfusion-induced damage via inhibition of p38 MAPK to NF-*κ*B signalling. *Pharmacological Research*.

[51] Lian M., Sun Y., Lin Y. (2017). p-JAK2 plays a key role in catalpol-induced protection against rat intestinal ischemia/reperfusion injury. *RSC Advances*.

[52] Montero M. F., Saurim R., Bonservizi W. G. S., Koike M. K., Taha M. O. (2014). Heart injury following intestinal ischemia reperfusion in rats is attenuated by association of ischemic preconditioning and adenosine. *Acta Cirurgica Brasileira*.

[53] Kim J. H., Kim J., Chun J., Lee C., Im J. P., Kim J. S. (2018). Role of iRhom2 in intestinal ischemia-reperfusion-mediated acute lung injury. *Scientific Reports*.

[54] Saidi S. A., Ncir M., Chaaben R., Jamoussi K., van Pelt J., Elfeki A. (2017). Liver injury following small intestinal ischemia reperfusion in rats is attenuated byPistacia lentiscusoil: antioxidant and anti-inflammatory effects. *Archives of Physiology and Biochemistry*.

[55] Alexandropoulos D., Bazigos G. V., Doulamis I. P. (2017). Protective effects of N-acetylcystein and atorvastatin against renal and hepatic injury in a rat model of intestinal ischemia-reperfusion. *Biomedicine & Pharmacotherapy = Biomedecine & Pharmacotherapie*.

[56] Hsieh Y.-H., McCartney K., Moore T. A. (2011). Intestinal ischemia-reperfusion injury leads to inflammatory changes in the brain. *Shock (Augusta, Ga.)*.

[57] He G.-Z., Zhou K.-G., Zhang R., Wang Y. K., Chen X. F. (2012). Impact of intestinal ischemia/reperfusion and lymph drainage on distant organs in rats. *World Journal of Gastroenterology*.

[58] Schwarz B., Salak N., Hofstötter H. (1999). Intestinal ischemic reperfusion syndrome: pathophysiology, clinical significance, therapy. *Wiener Klinische Wochenschrift*.

[59] Pan W. Z., Du J., Zhang L. Y., Ma J. H. (2018). The roles of NF-kB in the development of lung injury after one-lung ventilation. *European Review for Medical and Pharmacological Sciences*.

[60] Kumar D., Singla S. K., Puri V., Puri S. (2015). The restrained expression of NF-kB in renal tissue ameliorates folic acid induced acute kidney injury in mice. *PLoS One*.

[61] Shetty S. K., Tiwari N., Marudamuthu A. S. (2017). p53 and miR-34a feedback promotes lung epithelial injury and pulmonary fibrosis. *The American Journal of Pathology*.

[62] Yano T., Abe K., Tanno M. (2018). Does p53 inhibition suppress myocardial ischemia-reperfusion injury?. *Journal of Cardiovascular Pharmacology and Therapeutics*.

[63] Bu Y., Li X., He Y. (2016). A phosphomimetic mutant of RelA/p65 at Ser536 induces apoptosis and senescence: an implication for tumor-suppressive role of Ser536 phosphorylation. *International Journal of Cancer*.

[64] Chen S., Jiang S., Zheng W. (2017). RelA/p65 inhibition prevents tendon adhesion by modulating inflammation, cell proliferation, and apoptosis. *Cell Death & Disease*.

[65] Chillemi G., Kehrloesser S., Bernassola F. (2017). Structural evolution and dynamics of the p53 proteins. *Cold Spring Harbor Perspectives in Medicine*.

[66] Pillai A. K., Kalva S. P., Hsu S. L. (2018). Quality improvement guidelines for mesenteric angioplasty and stent placement for the treatment of chronic mesenteric ischemia. *Journal of Vascular and Interventional Radiology*.

[67] Klar E., Rahmanian P. B., Bücker A., Hauenstein K., Jauch K. W., Luther B. (2012). Acute mesenteric ischemia: a vascular emergency. *Deutsches Ärzteblatt International*.

[68] Luther B., Mamopoulos A., Lehmann C., Klar E. (2018). The ongoing challenge of acute mesenteric ischemia. *Visceral medicine*.

[69] Terlouw L. G., Moelker A., Abrahamsen J. (2020). European guidelines on chronic mesenteric ischaemia - joint United European Gastroenterology, European Association for Gastroenterology, Endoscopy and Nutrition, European Society of Gastrointestinal and Abdominal Radiology, Netherlands Association of Hepatogastroenterologists, Hellenic Society of Gastroenterology, Cardiovascular and Interventional Radiological Society of Europe, and Dutch Mesenteric Ischemia Study group clinical guidelines on the diagnosis and treatment of patients with chronic mesenteric ischaemia. *United European Gastroenterology Journal*.

[70] Tilsed J. V. T., Casamassima A., Kurihara H. (2016). ESTES guidelines: acute mesenteric ischaemia. *European Journal of Trauma and Emergency Surgery*.

[71] Silvestri L., van Saene H. K. F., Zandstra D. F., Marshall J. C., Gregori D., Gullo A. (2010). Impact of selective decontamination of the digestive tract on multiple organ dysfunction syndrome: systematic review of randomized controlled trials∗. *Critical Care Medicine*.

[72] Wong P. F., Gilliam A. D., Kumar S., Shenfine J., O'Dair G. N., Leaper D. J. (2005). Antibiotic regimens for secondary peritonitis of gastrointestinal origin in adults. *Cochrane Database of Systematic Reviews*.

[73] Wyers M. C. (2010). Acute mesenteric ischemia: diagnostic approach and surgical treatment. *Seminars in Vascular Surgery*.

[74] Hagiya H., Kokado R., Ueda A. (2019). Association of adverse drug events with broad-spectrum antibiotic use in hospitalized patients: a single-center study. *Internal Medicine*.

[75] Stine J. G., Lewis J. H. (2013). Hepatotoxicity of antibiotics: a review and update for the clinician. *Clinics in Liver Disease*.

[76] Morales-Alvarez M. C. (2020). Nephrotoxicity of antimicrobials and antibiotics. *Advances in Chronic Kidney Disease*.

[77] Nabavi S., Di Lorenzo A., Izadi M., Sobarzo-Sánchez E., Daglia M., Nabavi S. (2015). Antibacterial effects of cinnamon: from farm to food, cosmetic and pharmaceutical industries. *Nutrients*.

[78] Vasconcelos N. G., Croda J., Simionatto S. (2018). Antibacterial mechanisms of cinnamon and its constituents: a review. *Microbial Pathogenesis*.

